# Application of maltose as energy source in protein-free CHO-K1 culture to improve the production of recombinant monoclonal antibody

**DOI:** 10.1038/s41598-018-22490-8

**Published:** 2018-03-06

**Authors:** Dawn Sow Zong Leong, Brian Kah Hui Teo, Janice Gek Ling Tan, Hayati Kamari, Yuan Sheng Yang, Peiqing Zhang, Say Kong Ng

**Affiliations:** Bioprocessing Technology Institute, Agency for Science, Technology and Research (A*STAR), Singapore, Singapore

## Abstract

Oligosaccharides are generally considered to be un-utilized for growth of mammalian cells because their permeability across the cell membrane is low. However, in our previous study, we discovered that CHO and HEK293 cells consume maltose in culture media without serum and glucose. This is interesting because the transporter for maltose in mammalian cells has not been discovered to-date, and the only animal disaccharide transporter that is recently discovered is a sucrose transporter. The application of oligosaccharides in mammalian cell-based biopharmaceutical manufacturing can be beneficial, because it can theoretically increase carbohydrate content of the culture medium and decrease lactate production. Here, we first determined that specific maltose consumption rate in CHO cells was similar to galactose and fructose at 0.257 ng/cell/day. We then demonstrated that CHO cells can be cultivated with reasonable cell growth using higher maltose concentrations. After which, we evaluated the use of maltose supplementation in the production of a recombinant monoclonal antibody in batch and fed-batch cultures, demonstrating improvements in recombinant monoclonal antibody titer of 15% and 23% respectively. Finally, glycosylation profiles of the antibodies were analyzed.

## Introduction

Mammalian cell cultures are commonly used for the manufacturing of recombinant glycoprotein products because of its ability to fold complex proteins and to add human-like glycans to glycoproteins. In the manufacturing of such products for biopharmaceutical applications, mammalian cell culture that is animal component-free and serum-free is recommended by regulatory authorities, to avoid potential pathogenic contaminants from animal-derived products used to cultivate the cells. In these serum-free cell cultures, glucose is commonly used as the carbohydrate source, since it can be effectively transported through the phospholipid bilayer cell membrane via the glucose transporters (GLUT) and sodium-glucose linked transporters (SGLT)^1^. Other carbohydrates that have been reported to be consumed by the cells in serum-free cell culture are monosaccharides: galactose, fructose and mannose^2^. The use of oligosaccharides in mammalian cell cultures has only been reported in serum-supplemented cultures because serum saccharidases break down these oligosaccharides in the culture medium for utilization by the cells^3^.

For practical applications, the use of oligosaccharides for mammalian cell cultivation can be advantageous to the serum free suspension cell culture of transformed cell lines typically used for the biomanufacturing of recombinant protein therapeutics. Two glucose-related issues are commonly encountered for such cell cultures: Firstly, glucose is commonly the limiting substrate in serum-free suspension cell batch culture due its high consumption rate and the high cell density attained in suspension cell cultures. This is despite the high initial glucose concentration in the culture media: glucose is commonly the most abundant nutrient in most media formulations for mammalian cell batch culture^4^, at 2 to 10 fold higher concentration than the next most abundant nutrient utilized by the cells during cell growth. Further loading of the cell culture media with glucose is limited by the overall osmolality of the culture media since hyperosmotic culture media has been shown to be detrimental to cell growth^5,6^. Secondly, glucose is known to contribute to high lactate levels in culture, since these transformed cell lines have high rates of glycolysis and lactate production, a phenotype described as the Warburg effect^7^. This becomes a productivity limitation to both batch and fed-batch cultures, because lactate is toxic to the cells and increased lactate concentrations in the bioreactor will result in decreased cell growth rate^8,9^.

The use of oligosaccharides in serum free suspension cell culture may potentially address both issues: As oligosaccharides contribute to lower osmolality per unit mass concentration, these sugars can potentially increase the sugar availability to cells in batch culture media since higher mass concentrations can be used. Depending on the rate of conversion of the oligosaccharides to monosaccharides, it may also mitigate lactate accumulation in the bioreactor by providing a source of sugars that is not readily available to the cells, thereby limiting glycolysis and lactate production. In theory, this will be somewhat similar to maintaining low glucose concentrations in bioreactors as achieved by dynamic online feeding strategies^10^, albeit being practically simpler to set up and implement.

Recently, we reported our discovery that mammalian cells consume maltose, a disaccharide, in serum-free cell culture: we demonstrated that CHO and HEK293 cells can be adapted to serum-free culture media containing maltose as the sole carbohydrate source, intracellular maltose was detected in maltose-supplemented cell culture, and that maltose in a protein-free chemically-defined medium is only depleted in the presence of cells^11^. This is somewhat surprising because currently, the only animal disaccharide transporter that has been reported is a sucrose transporter in *Drosophila melanogaster*^12^.

While the mechanism of this phenomenon is still not well-understood, we investigated the kinetics and application of maltose in monoclonal antibody production in this study. A Monod model for specific maltose consumption was first proposed, leading to the evaluation of different maltose concentrations to improve the growth of CHO-K1 cultures adapted to maltose-only medium. Applications of maltose supplementation in batch and fed-batch culture of an antibody producing CHO-K1 cell line was then studied.

## Results

### Characterization of maltose metabolism kinetics

**Figure 1:**
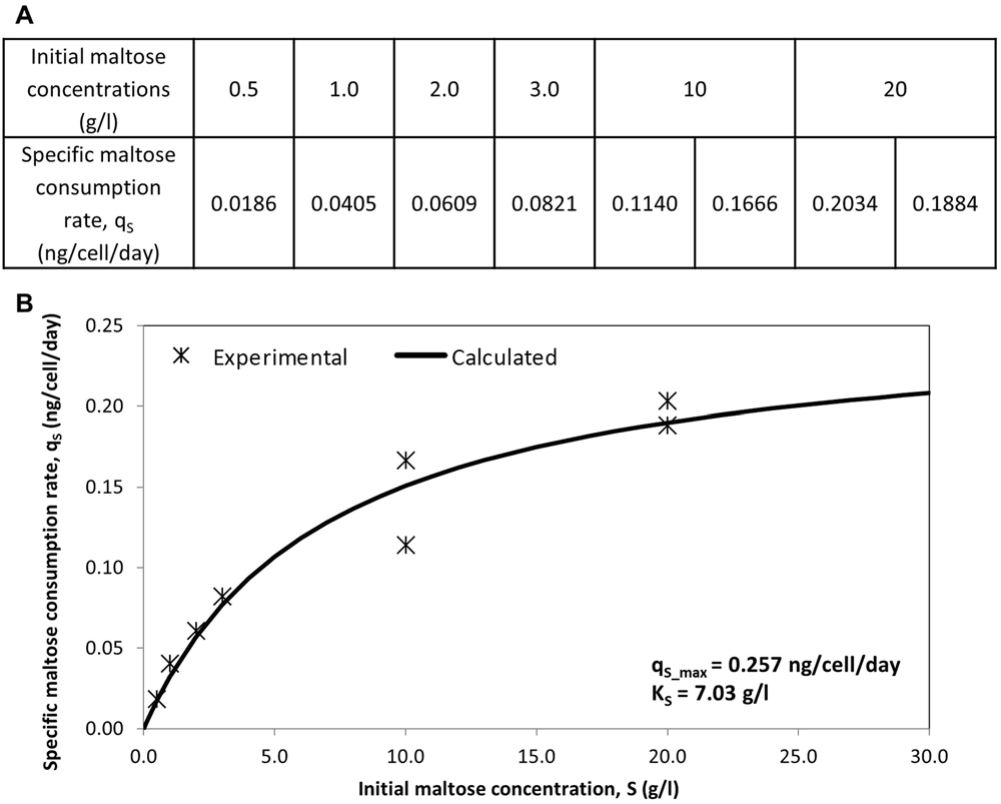
Monod model for specific maltose consumption rate. (**A**) Specific maltose consumption rates for cultures with different initial maltose concentrations from different experiments were determined as the slope from the plots of maltose concentrations against cumulative integral viable cell densities (IVCD), according to Equation 4. (**B**) Maximum specific maltose consumption rate (q_s_max_) and affinity constant (K_s_) were determined by non-linear regression, according to Equation 5. The Monod model for specific maltose consumption rate calculated using these parameters and the experimental data used for the model were then plotted.

To characterize the kinetics of maltose metabolism, SH87, a CHO-K1 cell line that produces an anti-Her2 monoclonal antibody^13^ was cultured in a protein free chemically defined medium (PFCDM) containing 4 g/l glucose supplemented with 0.5, 1, 2, 3, 10 or 20 g/l maltose, which gave maximum viable cell densities greater than 8 × 10^6^ cells/ml. The specific maltose consumption rates were obtained from plots of maltose concentrations against cumulative integral viable cell densities (IVCD) according to Equation 4. These plots gave straight trendlines with R^2^ values between 0.853 and 0.999 when 3 to 5 data points after glucose depletion were used (Supplementary Fig. 1), suggesting that the specific maltose consumption rates were fairly constant when maltose was metabolized. The slopes of these trendlines gave the average specific maltose consumption rates of these cultures and are tabulated in Fig. 1A.

The specific maltose consumption rates increased with increasing initial maltose concentrations, verifying the concentration dependence of maltose metabolism at these concentrations. Additionally, it was observed that the magnitude of increase in specific consumption decreased at higher initial maltose concentrations, suggesting that there may be a maxima in this relationship. As such, the data was fitted to a Monod model according to Equation 5 by non-linear regression. The data fitted to the model (Fig. 1B) to obtain a maximum specific maltose consumption rate (q_s_max_) of 0.257 ng/cell/day and an affinity constant (K_s_) of 7.03 g/l. Comparing with published specific monosaccharide consumption rates using 3.6 g/l of monosaccharides^2^, the maximum specific maltose consumption rate determined here is similar to the specific consumption rates of fructose (0.21 ng/cell/day) and galactose (0.21 ng/cell/day) while being lower than the measured specific consumption rates of glucose (0.76 ng/cell/day) and mannose (0.88 ng/cell/day). This suggests that maltose can be metabolized at rates similar to fructose and galactose while maltose metabolism will be slower than that with glucose and mannose. As such, we described a first estimate of mammalian cell maltose metabolism kinetics that has not been previously reported.

**Figure 2:**
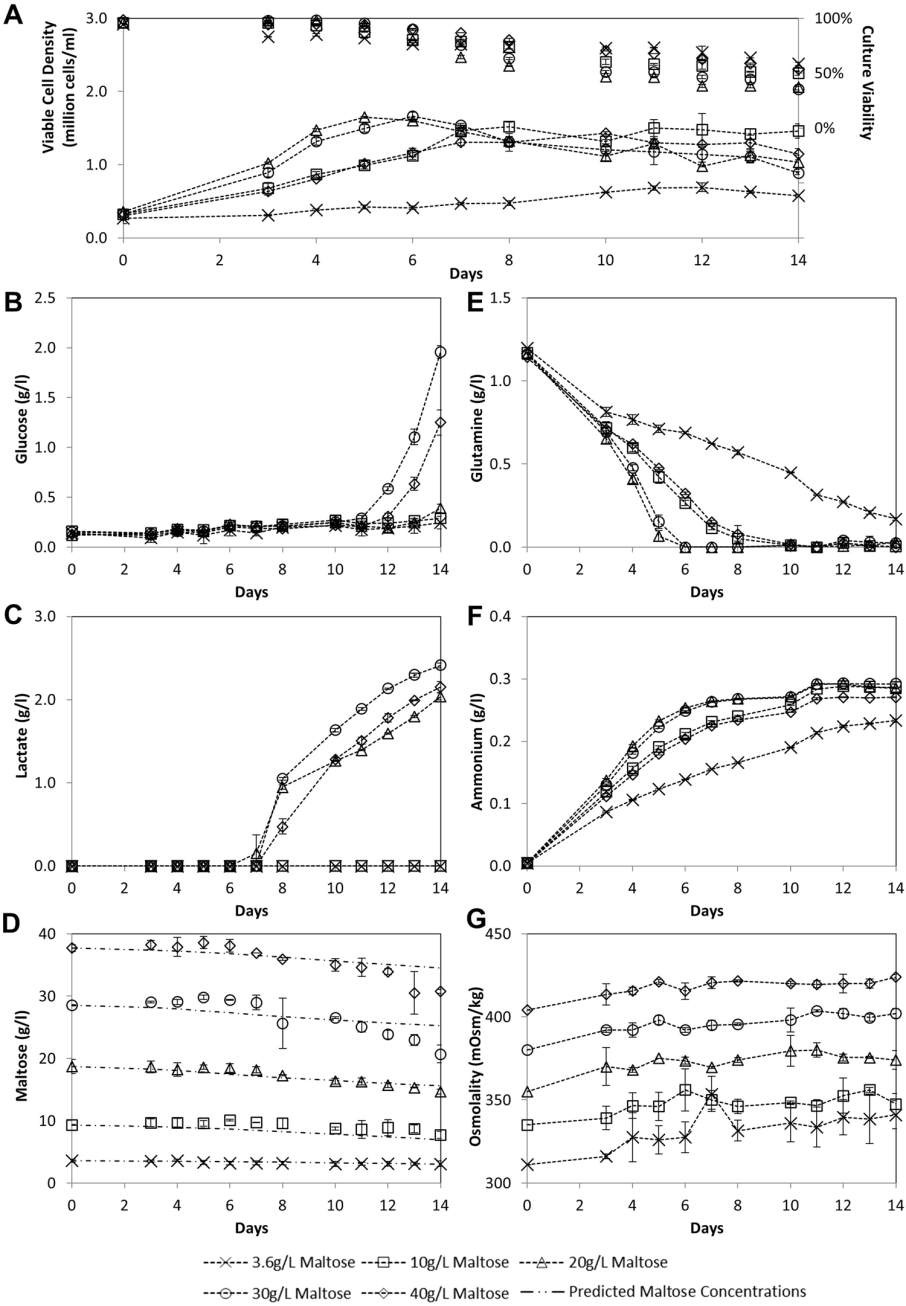
Data from CHO-K1 culture with increasing concentration of maltose. CHO-K1 cells adapted to PFCDM with 10 g/L of maltose was cultivated in PFCDM with 3.6, 10, 20, 30 and 40 g/L maltose in duplicate batch shake flask cultures. (**A**) Viable cell densities (lined marker) and culture viabilities (marker only), (**B**) Glucose, (**C**) Lactate, (**D**) Actual maltose (marker only) and calculated maltose (line only), (**E**) Glutamine, (**F**) Ammonium, and (**G**) Osmolality profiles. To determine calculated maltose concentrations, specific maltose consumption was first determined using Monod model (Equation 5), calculated maltose concentrations were then determined based on IVCD using Equation 4.

Based on this model, we hypothesized that the CHO-K1 cells adapted to propagate in serum free medium with maltose as sole carbohydrate source should be able to proliferate faster with increasing maltose concentrations. We therefore evaluated the growth of these cells in PFCDM medium without glucose but supplemented with 3.6, 10, 20, 30 and 40 g/l maltose (Fig. 2). Exponential specific growth rates of the cultures were calculated using viable cell density data up till Day 5 according to Equation 1, to give 0.004, 0.0101, 0.0140, 0.0139 and 0.0107 h^−1^ respectively. Compared to CHO cells cultivated in normal medium with glucose which typically has a specific growth rate of about 0.03 h^−1^, this slower growth corroborates with the lower specific maltose consumption discussed above, and suggests that carbohydrate metabolism may be limiting in these maltose cultures. More importantly, the cells indeed proliferated faster with increasing maltose concentrations from 3.6 g/l to 20 and 30 g/l (Fig. 2A) to reach maximum viable cell density (VCD) of 1.6 × 10^6^ cells/ml. Further increase in maltose concentration to 40 g/l gave a growth curve similar to the 10 g/l culture. This can be attributed to its initial high osmolality at 404 mOsm/kg (Fig. 2G), countering the effects of faster maltose metabolism.

Essential biochemicals in culture supernatant were also analyzed (Fig. 2B–F). From Fig. 2B, it is observed that glucose concentrations of all cultures remained below the instrument lower limit of 0.20 g/l up till Day 10. As cell proliferation was observed up to Day 7, this demonstrates that residual glucose could not have been responsible for the observed cell growth, and that maltose was likely used as an alternative carbohydrate source. From Day 11 onwards, glucose concentrations increased for the 30 g/l and 40 g/l cultures to 1.96 g/l and 1.25 g/l respectively, suggesting that a small percentage (6.5% and 3.1% respectively) of maltose was broken down: We postulate that there may be release of transiently active maltase from lysed cells during death phase, and the high maltose concentrations in these cultures may have resulted in faster and more noticeable breakdown of maltose to glucose in these cultures compared to cultures with lower maltose concentrations.

As for lactate, it was not detectable until Day 7 (Fig. 2C). We postulate that the delayed accumulation of lactate could be attributed to a reduced Warburg effect^7^ as a result of substituting glucose with maltose. This change in carbohydrate source may have compelled the cells to be more energy efficient instead of being dependent on glycolysis. From Day 7, lactate was only detected for the 20, 30 and 40 g/l cultures and not for the 3.6 and 10 g/l cultures. Day 7 also coincided with the cultures entering death phase. As such, we postulate that the cultures with higher maltose concentrations may have more glycolysis intermediates that were processed to lactate when the cells die.

From Fig. 2A,E and F, we observed a direct correlation between cell growth, glutamine consumption and ammonium production: cultures with faster growth had faster glutamine consumption and ammonium production. When specific rates were determined according to Equation 4, specific glutamine consumption were 0.795, 0.855, 1.431, 1.481 and 0.630 ng/cell/day respectively, and specific ammonia production prior to glutamine depletion were 1.399, 1.178, 1.863, 2.035 and 1.264 ng/cell/day respectively, corroborating with the observed correlation and suggesting that more glutamine was used to provide energy to support the faster cell growth observed in the 20 g/l and 30 g/l maltose cultures. These specific rates were also higher than the maximum maltose specific consumption rate of 0.257 ng/cell/day, and glutamine depletion coincided with peak VCD. These suggest that glutamine (and potentially other amino acids) is likely a main energy source in these cultures leading to ammonium production, and maltose provided an additional and limiting energy stream to allow the cells to proliferate in the absence of glucose.

Lastly, maltose utilization was observed to increase with increasing maltose concentrations (Fig. 2D). Calculated maltose concentrations based on our Monod model (Fig. 1) were also plotted (Fig. 2D), demonstrating that the observed maltose concentrations were in-line with calculated values.

Taken together, this data confirms that CHO-K1 cells can proliferate in maltose-only medium without serum, hydrolysates or protein supplements to cell density greater than 10^6^ cells/ml. This also demonstrates that high maltose concentrations up to 30 g/L can be used for batch CHO cell culture without detrimental effects due to osmolality. As such, these results suggest that maltose can be used as a glucose-replacement in routine cultivation of adapted mammalian cell lines in serum-free media at a lower growth rate.

### Comparison of batch cultures with high glucose and maltose concentrations

**Figure 3:**
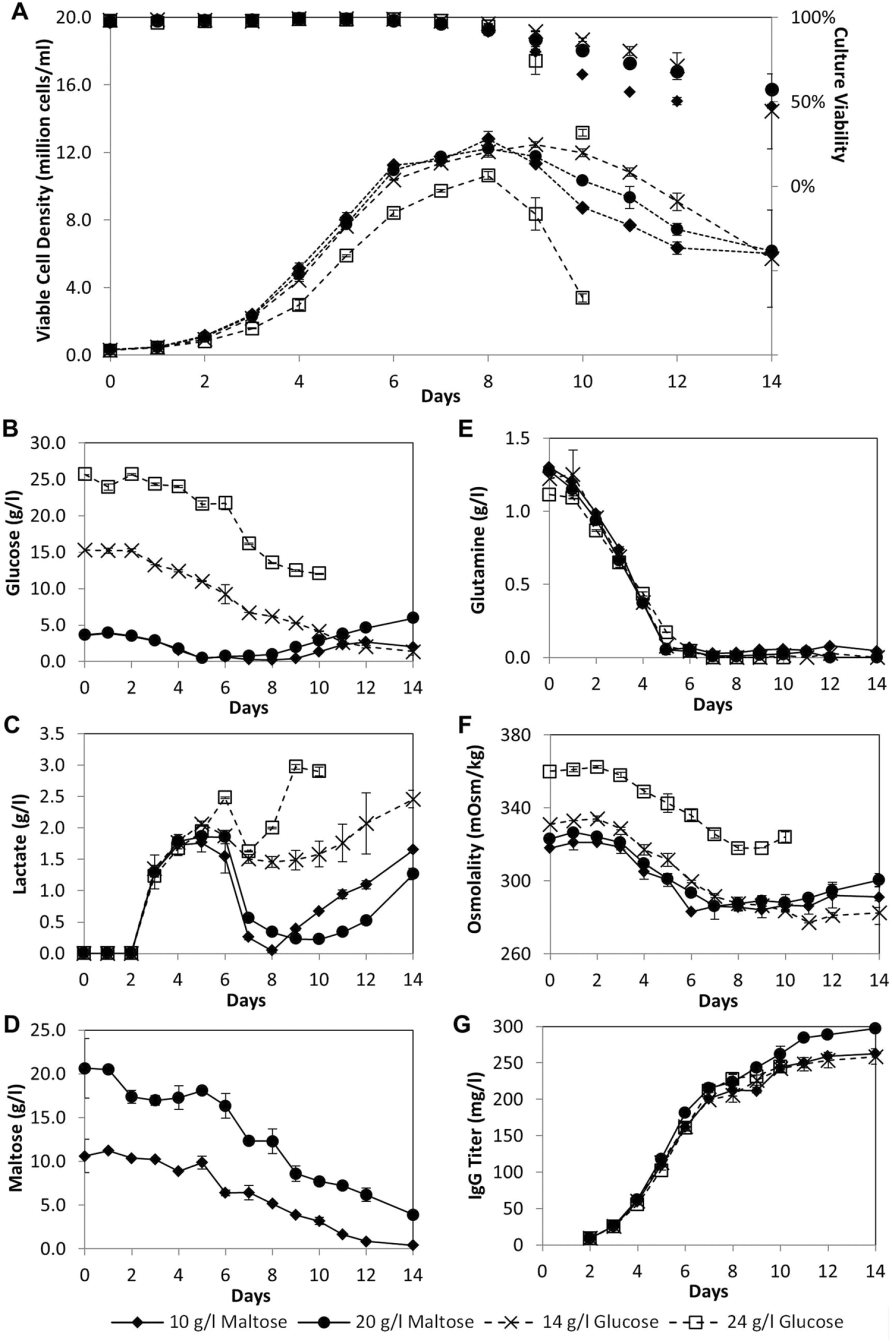
Data from SH87 culture with high concentrations of glucose and maltose. SH87 cells normally cultured in glucose-only PFCDM were seeded into PFCDM with 14 g/l glucose, 24 g/l glucose, 4 g/l glucose + 10 g/l maltose, or 4 g/l glucose + 20 g/l maltose. (A) Viable cell densities (lined marker) and culture viabilities (marker only), and (**B**) Glucose, (**C**) Lactate, (**D**) Maltose, (**E**) Glutamine, (**F**) Osmolality, and (**G**) IgG titer profiles. For maltose profile, averages of 2 technical replicates from one set of shake flasks were plotted. Averages from 2 replicate shake flasks were used for other graphs.

To study the effect of supplementing maltose at high concentrations in batch production of antibodies, we compared the culture profiles of SH87 in media supplemented with 4 g/l glucose and 10 g/l or 20 g/l maltose, to cultures of the same cells in media containing 14 g/l and 24 g/l glucose (Fig. 3). The 24 g/l glucose culture grew slower, attained a lower maximum VCD of 10.6 × 10^6^ cells/ml, and reached a low viability of less than 50% faster than the other 3 cultures (Fig. 3A). The slower growth of the 24 g/l glucose culture can be attributed to the higher osmolality (360 mOsm/kg) of the culture medium (Fig. 3F), while the culture media of the other 3 cultures had similar osmolality between 318 and 331 mOsm/kg that were in the optimum pH range for the cultivation of mammalian cells. This confirms a limitation of high glucose loading in batch culture medium because glucose contributes to osmolality significantly and can affect cell growth at high concentrations. In contrast, the same mass concentrations of maltose contributed to less increase in osmolality and thus had no negative effect on cell growth.

Comparing the growth profiles of the 10 g/l maltose, 20 g/l maltose and 14 g/l glucose cultures (Fig. 3A), they were similar even after glucose concentration reached its minimal on Day 5 for the maltose supplemented cultures (Fig. 3B). This suggests that maltose metabolism is not restricting cell growth, in contrast to previous observation of slower growth observed at maltose concentrations lower than 3 g/l after glucose depletion^11^. The hypothesis of a higher maltose metabolism at these concentrations is supported by observations of glucose accumulation in the 10 g/l and 20 g/l maltose supplemented cultures (Fig. 3B), a phenomenon not observed in our previous study^11^. This suggests that maltose was hydrolyzed faster than the cells’ metabolic requirements during the corresponding culture times: Glucose accumulation was observed in the 20 g/l maltose supplemented culture after Day 5, when the cells were still growing at a similar rate as the 14 g/l glucose culture, demonstrating that maltose metabolism was not limiting cell growth. At lower maltose concentration of 10 g/l, glucose accumulation was observed only after Day 8, when the cells are entering death phase. With the cells growing similarly to the 14 g/l glucose culture, this indicates that maltose metabolism was just ample to support cell growth at 10 g/l maltose without excess glucose accumulating in the culture medium.

Also worth noting here is that the lactate consumption in the glucose only cultures were minimal throughout the culture duration, in contrast to the maltose supplemented cultures which consumed most of the lactate from Days 5 to 8 (Fig. 3C). While a second lactate production phase was observed after Days 9 and 10 for the 10 g/l and 20 g/l maltose supplemented cultures respectively, the lactate concentrations in these cultures reached significantly lower levels as compared to the glucose cultures. This verifies another limitation of glucose loading in batch culture medium: because lactate accumulation can result in cell toxicity^8,9^, the lack of its consumption with high glucose loading can limit the growth of the cell culture. In this case, this lactate toxicity may have also contributed to the faster death phase observed for the 24 g/l glucose culture. Ammonium production profiles were similar for all 4 cultures (data not shown) because these cells were cultivated in the presence of glucose, and as such, ammonium is unlikely a contributing factor to growth and production differences in this experiment.

Examining the maltose profiles of the maltose supplemented cultures, maltose consumption were faster after glucose depletion on Day 5 (Fig. 3D), similar to previous data^11^, and maltose was not depleted for both maltose supplemented cultures till the end of the run on Day 14. Similarly, glucose was not depleted for both glucose cultures (Fig. 3B). While sugars were present in excess, other nutrients may be limiting the growth of these cells, for example, glutamine was used up at Day 6 for all 4 cultures (Fig. 3E). These other limitations may have affected growth similarly to cause the similar growth profiles of the maltose supplemented cultures and the 14 g/l glucose culture.

With the similar growth profiles, it was interesting to note that the 20 g/l maltose supplemented culture resulted in a maximum IgG titer of 298 mg/l, 15% higher than that from the 14 g/l glucose culture and 10 g/l maltose supplemented cultures which gave maximum titers of 259 mg/l and 263 mg/l respectively (Fig. 3G). To examine this, the specific IgG productivities of the cultures were determined according to Equation 3 (Table 1 and Supplementary Fig. 2). We noted that the specific IgG productivities for all 4 cultures decreased after Day 7, when glutamine concentrations reached their minimum, suggesting that glutamine may be limiting IgG productivity from Day 7. Regardless of glutamine limitation, we observed that specific IgG productivities were 21% to 29% higher in the culture with more glucose or with more maltose, suggesting that higher sugar concentrations can improve specific IgG productivities. When we compared the specific IgG productivities of maltose supplemented cultures with their corresponding glucose culture having the same total sugar concentration, the maltose supplemented cultures had 13% to 17% lower specific IgG productivities up to Day 6, in concurrence with the previous observations that maltose metabolism is less efficient than that of glucose. However, specific IgG productivities of maltose supplemented cultures became 25% to 26% higher than specific IgG productivities of their corresponding glucose culture from Day 7, after glutamine depletion and the cultures reached stationary phase, even though sugars were not limiting. As this cannot be accounted for by differences in IVCD profile (data not shown), we postulate that the higher lactate levels in the glucose cultures may have negatively impacted the IgG productivities from Day 7. As a result, the 20 g/l maltose supplemented culture had the highest maximum IgG titer of 297 mg/l, which is respectively 15% and 21% higher than that achieved in the 14 g/l and 24 g/l glucose cultures, despite similar growth profiles.

**Table 1:** Specific IgG productivities of SH87 cultivated in PFCDM with high concentrations of glucose and maltose. SH87 cells normally cultured in glucose-only PFCDM were seeded into PFCDM with 14 g/l glucose, 24 g/l glucose, 4 g/l glucose + 10 g/l maltose, or 4 g/l glucose + 20 g/l maltose. IgG titers were then plotted against IVCD to obtain the specific IgG productivity as the slope of the graphs, according to Equation 3.

Culture media sugar content	Specific IgG productivity up to Day 6 (pg/cell/day)	Specific IgG productivity from Day 7 (pg/cell/day)
14 g/l glucose	7.74	0.96
24 g/l glucose	10.0	1.17
4 g/l glucose + 10 g/l maltose	6.73	1.21
4 g/l glucose + 20 g/l maltose	8.29	1.47

### Application of maltose in fed-batch cultures

**Figure 4:**
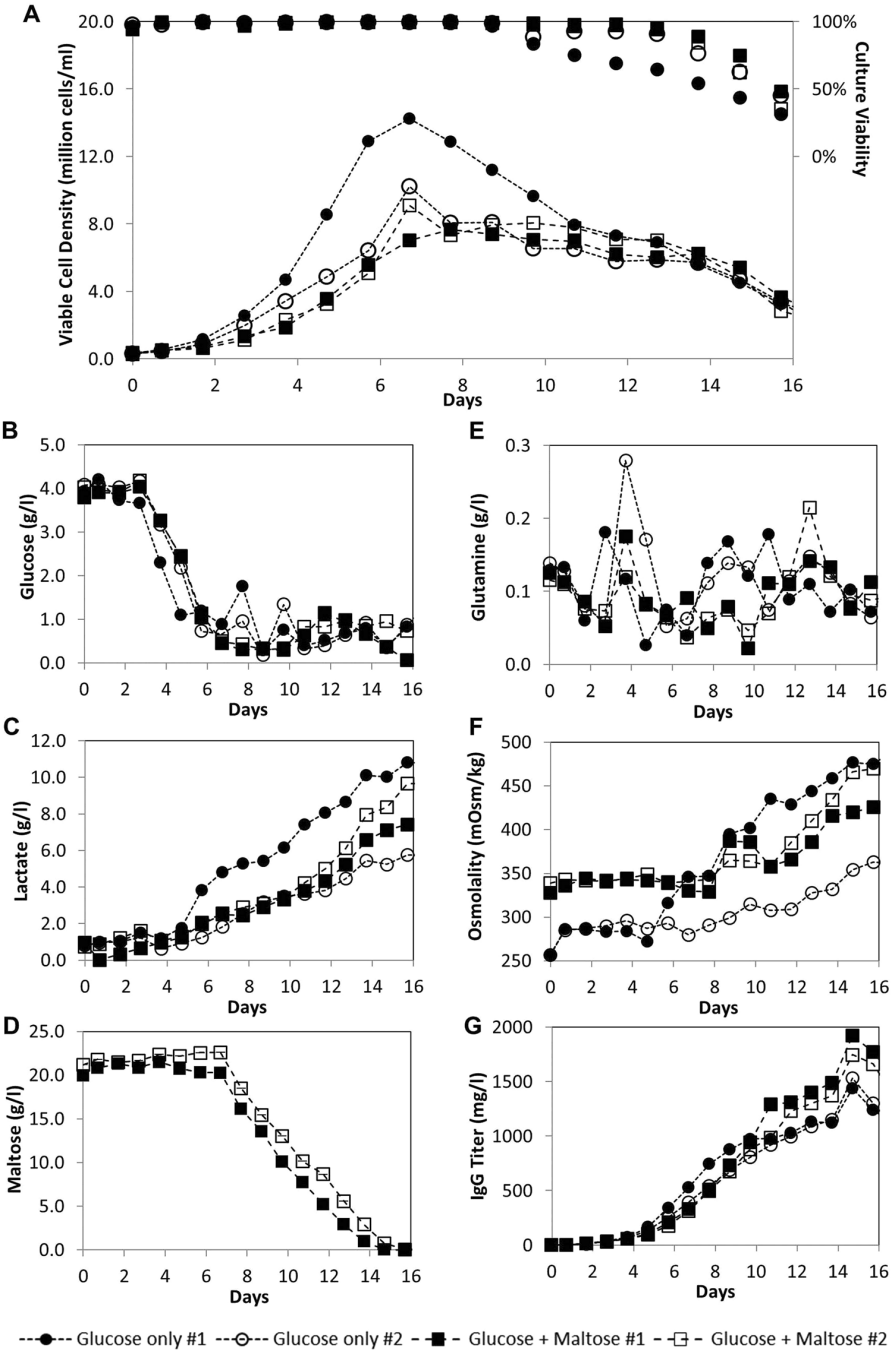
Data from SH87 fed-batch bioreactor cultures in glucose only or glucose + maltose base medium. SH87 cells normally cultured in glucose-only PFCDM were seeded into PFCDM with 4 g/l glucose or 4 g/l glucose + 20 g/l maltose in 2 liter stirred tank bioreactors. The cultures with glucose only base medium were fed daily with 100% of its calculated glucose requirement while the cultures with glucose and maltose base medium was fed daily with 50% of its calculated glucose requirement. Other nutrients were fed similarly in a separate feed using glutamine as reference nutrient. (**A**) Viable cell densities (lined marker) and culture viabilities (marker only), (**B**) Glucose, (**C**) Lactate, (**D**) Maltose, (**E**) Glutamine, (**F**) Osmolality, and (**G**) IgG titer profiles. Duplicate bioreactor cultures were performed for each fed-batch conditions and data from all 4 cultures are plotted.

Since fed-batch culture is a popular mode for the manufacturing of monoclonal antibodies, we evaluated the use of maltose in fed-batch cultures. In contrast to batch cultures where initial glucose concentration is limited by the detrimental effect on cell growth due to consequent increase in osmolality beyond a certain limit, fed-batch cultures do not have this limitation since glucose can be fed continually into the cultures. As such, we investigated whether maltose can be used to supplement glucose in fed-batch cultures to drive the cells towards a slower but more efficient metabolism: Using a common glucose concentration setpoint of 0.5 g/l, SH87 in media supplemented with 4 g/l glucose and 20 g/l maltose was fed daily with 50% of its calculated glucose requirement, while cultures of the same cells in media containing 4 g/l glucose was fed daily with 100% of its calculated glucose requirement. Duplicate bioreactor cultures were performed for each condition and the growth, biochemical and IgG titer profiles of these cultures were plotted in Fig. 4.

In contrast to the lactate consumption observed in the batch cultures (Fig. 3), there was no lactate consumption in the fed-batch cultures, even in the maltose-supplemented cultures with a 50% glucose feed (Fig. 4C). We postulate that this may be due to the consistent nutrient feeding in the fed-batch cultures, whereas glucose, glutamine or other nutrients were depleted in the batch cultures to result in the observed lactate consumption. Nonetheless, IgG titers of fed-batch cultures (Fig. 4G) were more than 5 fold that obtained from the batch cultures (Fig. 3G), demonstrating that the feeding strategy was successful in improving IgG production as expected of fed-batch cultures.

Comparing between the fed-batch cultures, the VCD, culture viabilities and lactate plots of the maltose-supplemented cultures and Glucose-only #2 culture were similar, while Glucose-only #1 culture had a higher maximum VCD, a faster decrease in culture viability and a faster lactate accumulation (Fig. 4A and C). The faster increase in osmolality in Glucose-only culture #1 (Fig. 4F) is likely due to pH correction as a result of the faster lactate accumulation. These illustrate possible variability of these parameters in the SH87 fed-batch cultures. On the other hand, glucose and glutamine profiles of maltose-supplemented and glucose-only cultures were similar. In addition, IgG titer was fairly consistent between the replicate cultures despite the variability in VCD, lactate and osmolality observed in the glucose-only duplicate cultures.

Comparing the glucose profiles, it was interesting to note that despite being fed only at 50% of its calculated glucose requirement, the maltose-supplemented cultures maintained similar culture glucose concentrations as the glucose-only fed-batch cultures. Nonetheless, because of this reduced glucose feeding, specific glucose consumption of the maltose-supplemented cultures was 0.115 ± 0.007 ng/cell/day, 45% that of the glucose-only cultures at 0.254 ± 0.013 ng/cell/day (Table 2). These suggest that the maltose-supplemented cultures were most probably using a secondary energy source in addition to glucose to achieve comparable growth and culture viability profiles as Glucose-only #2 culture. This secondary energy source is likely to be maltose, because specific glutamine consumption rates were similar between the maltose-supplemented and glucose-only cultures (Table 2), and maltose consumption was observed in the maltose-supplemented cultures from Day 7, one day after glucose feeding was initiated in the maltose supplemented fed-batch cultures (Fig. 4D).

**Table 2:** Maximum IgG titers, specific production and consumption rates of SH87 in PFCDM with and without maltose supplement in fed-batch bioreactor cultures. SH87 cells normally cultured in glucose-only PFCDM were seeded in duplicate fed-batch bioreactor cultures (denoted by #1 and #2) as described in Fig. 4. Specific growth rates were determined according to Equation 1. Cumulative amounts of biochemicals produced by the cells were plotted against cumulative Integral Viable Cell (IVC) number to obtain the specific productivities as the slope of the graphs.

	Maltose supplement	#1	#2	Average ± Standard deviation
Maximum IgG titer (mg/l)	+	1920	1745	1833 ± 124
	−	1435	1530	1483 ± 67
Specific IgG production (pcd)	+	31.2	26.3	28.8 ± 3.5
	−	15.6	21.5	18.6 ± 4.1
Exponential specific growth rate (day^-1^)	+	0.49	0.501	0.496 ± 0.008
	−	0.696	0.607	0.652 ± 0.063
Specific glucose consumption (ng/cell/day)	+	0.11	0.119	0.115 ± 0.007
	−	0.263	0.245	0.254 ± 0.013
Specific glutamine consumption (ng/cell/day)	+	0.036	0.03	0.033 ± 0.004
	−	0.029	0.027	0.028 ± 0.001
Specific lactate production (ng/cell/day)	+	0.106	0.114	0.110 ± 0.005
	−	0.117	0.077	0.097 ± 0.028

With the maltose supplemented metabolism, we observed that there were 23% and 55% improvements in maximum IgG titers and specific IgG productivities at 1.833 ± 0.124 g/l and 28.8 ± 3.5 pcd, from the 1.483 ± 0.067 g/l and 18.6 ± 4.1 pcd observed in glucose-only fed-batch cultures respectively (Table 2). When compared to Glucose-only #2 culture with a more comparable growth profile which has maximum IgG titers and specific IgG productivities of 1.53 g/l and 21.5 pcd respectively, the improvements in maximum IgG titers and specific IgG productivities were 20% and 34% respectively.

One possible mechanism for the observed improvement in IgG production may be the higher initial osmolality of the maltose-supplemented cultures due to the additional 20 g/l maltose: This may have resulted in the 22% to 40% lower specific growth rates of the maltose-supplemented cultures compared to the glucose-only cultures (Table 2), to possibly allow slower and more productive maltose-supplemented cultures. Nonetheless, when we compared maltose-supplemented batch culture to glucose-only batch culture having similar initial osmolality (Fig. 3G), we noted that an improvement in IgG titer was similarly observed. This suggests that the improvement in maximum IgG titers observed in the maltose-supplemented fed-batch cultures is not only due to osmolality effect: We postulate that a lower availability of glucose, enabled by the presence of maltose, may have resulted in a more efficient cell metabolism in the maltose-supplemented culture, and this may have contributed to the observed higher maximum IgG titers.

To verify whether this lower availability of glucose can have the same effect in the absence of maltose, we compared SH87 glucose-only fed-batch cultures fed with 100% or 50% of their calculated glucose requirements in a separate experiment (Supplementary Fig. 3). While similar maximum VCD were reached in both conditions before glucose feeding was initiated, the culture viabilities of the glucose-only cultures with 50% calculated glucose feed dropped below 50% 4 days earlier than the cultures with 100% calculated glucose feed. Maximum IgG titers of the 50% fed cultures were also only 46.4% that of the 100% fed cultures. These confirmed that the presence of maltose enabled the use of a lower glucose feed to result in the observed higher maximum IgG titers in Fig. 4G.

**Figure 5:**
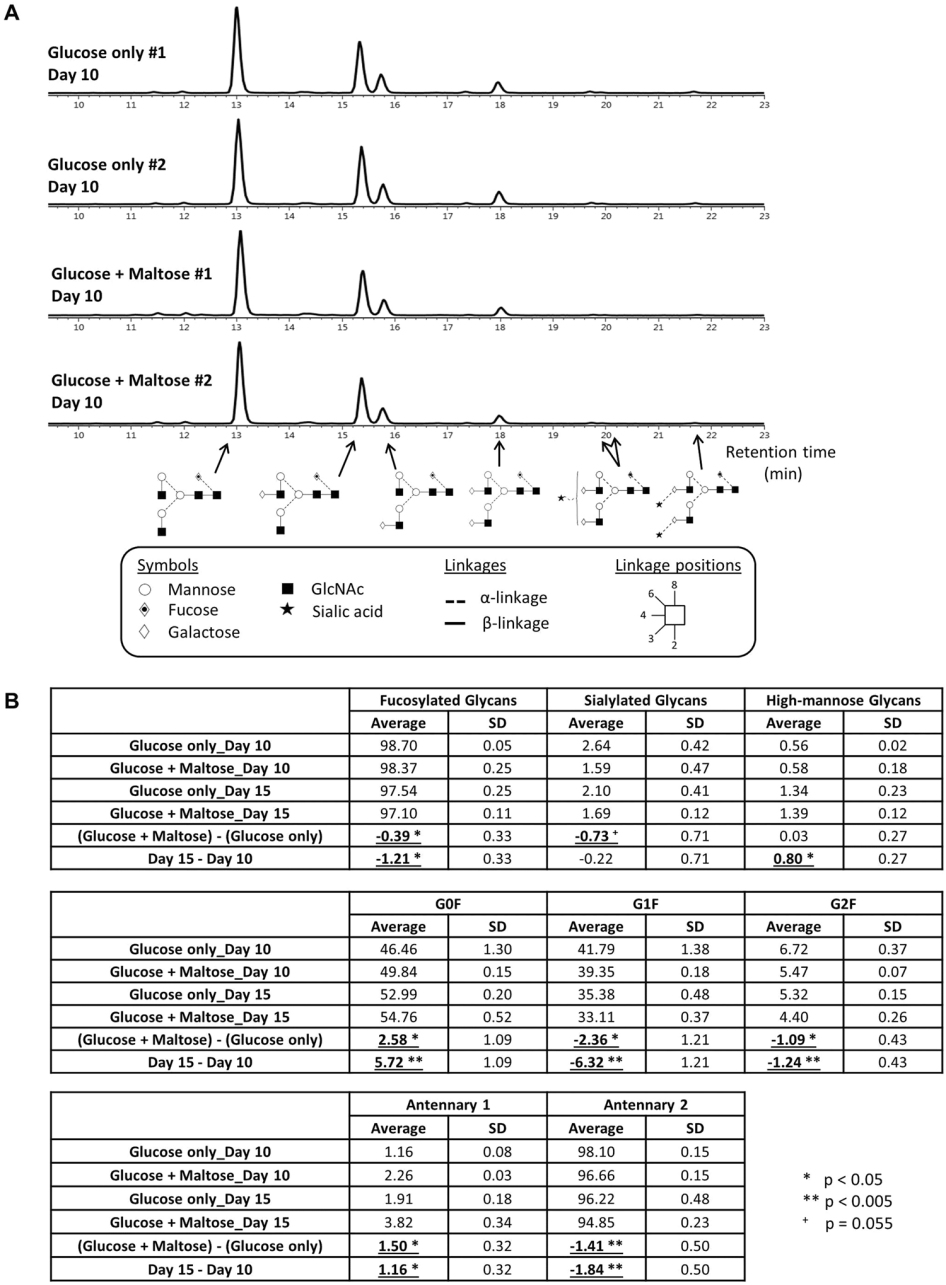
Glycosylation profiles of anti-Her2 antibody produced from SH87 fed-batch bioreactor cultures in glucose only or glucose + maltose PFCDM base medium. Anti-Her2 monoclonal antibodies were purified from samples from Day 10 and Day 15 of the SH87 fed-batch cultures in PFCDM with 4 g/l glucose or 4 g/l glucose + 20 g/l maltose (Fig. 4), and subjected to glycosylation profiling. (**A**) Representative fluorescence chromatograms of the four Day 10 samples with symbolic representations of selected structures. (**B**) Relative abundance of glycan structures. Average and standard deviation of the 2 biological replicates were shown. The average difference between Glucose + Maltose and Glucose only samples, as well as that between Day 15 and Day 10 samples were also calculated. Effects of maltose supplementation and late Day 15 harvest on the relative abundance of glycan structures were bold and underlined. p-values were calculated using paired one-tail Student’s t-Test on data from individual samples.

As glycosylation is a critical attribute of therapeutic IgG products, purified IgG from glucose-only and maltose supplemented cultures from Days 10 and 15 were subjected to glycosylation profiling (Fig. 5). The representative fluorescence chromatograms of Day 10 samples (Fig. 5A) showed that the glycosylation profiles of IgG from both glucose-only and maltose supplemented cultures were similar, suggesting that maltose supplementation did not grossly affect the glycan profiles of the monoclonal antibodies produced. When the relative abundance of glycan structures of the IgG from maltose supplemented cultures were compared to that from glucose-only cultures (Fig. 5B), we noted that there were marginally less fucosylated, sialylated, G1F, G2F and biantennary glycans, and more G0F and mono-antennary glycans. Most of these differences were also observed when glycans from Day 15 samples were compared to that from Day 10 samples, suggesting that maltose supplementation affected glycan profiles in a way that is mostly similar to a later harvest. The exceptions to the similarity are in the relative abundances of the sialylated and high-mannose glycans: while the later Day 15 harvests gave higher levels of high-mannose glycans and similar levels of sialylated glycans compared to Day 10 harvests, maltose supplementation gave lower levels of sialylated glycans and similar levels of high-mannose glycans compared to glucose-only cultures. This data suggests that maltose supplementation can also be used as a means to fine-tune monoclonal antibody glycosylation profile, especially in marginally reducing the sialylation level, which is known to improve antibody-dependent cellular cytotoxicity (ADCC) of therapeutic antibodies. Additionally, this may also be important in the field of biosimilar manufacturing, where matching of the biosimilar glycan profile to the innovator drug is an important criterion.

## Discussion

In this study, we proposed that the specific maltose consumption rates from SH87 maltose supplemented batch cultures can be fitted in a Monod model to obtain a maximum specific maltose consumption rate (q_s_max_) of 0.257 ng/cell/day and an affinity constant (K_s_) of 7.03 g/l. The model is then validated using maltose-adapted CHO-K1 cells cultured without glucose, serum or protein supplement, to a VCD greater than 10^6^ cells/ml. This demonstrates the utility of maltose as a glucose replacement candidate, although the cells will proliferate at a slower growth rate and to a lower maximum VCD. This is also the first published estimate of maltose metabolism kinetics in mammalian cells to our knowledge.

In addition, when culture media with both glucose and maltose were used and sugars were in excess, the practical application of maltose supplementation to increase the carbohydrate content of cell culture medium was demonstrated, since an increased glucose concentration is limited by the corresponding increase in osmolality. The utilization of maltose in batch cell culture has an added advantage of promoting lactate consumption, which will otherwise accumulate and become toxic to the cells. These factors contributed to a 15% improvement in the recombinant monoclonal antibody titer from batch cultures. We further demonstrated that maltose supplementation can also be applied to fed-batch bioreactor cultures to result in 23% and 55% improvements in maximum monoclonal antibody titers and specific monoclonal antibody productivities respectively, when compared to glucose-only fed-batch cultures. These results suggest that maltose supplementation can be applied as a simple and affordable culture media supplementation to improve monoclonal antibody yields in small scale batch processes and current manufacturing fed-batch processes.

Glycosylation profiling of the antibodies produced from the fed-batch cultures suggests that maltose supplementation marginally affected glycan profile similar to a later harvest, with an additional effect of slightly reducing sialylation levels without a concomitant increase in high-mannose glycans. This suggests that maltose supplementation can also be applied to marginally affect monoclonal antibody glycosylation profile, which is important in affecting the ADCC of the antibody therapeutic and in the physical matching of biosimilar glycan profile to that of the innovator drug.

## Methods

### Cell lines and propagation

CHO-K1 cells (American Type Culture Collection, Manassas, VA) adapted to maltose-only HyQ PF-CHO MPS (HyClone, Logan, UT)^11^ was further adapted into a DMEM/F12-based protein free chemically defined medium (PFCDM) supplemented with 10 g/L D-(+)-Maltose monohydrate (Sigma-Aldrich), 8 mM L-Glutamine (Sigma-Aldrich) and 0.1% Pluronic® F-68 (Life Technologies, Carlsbad, CA). An anti-Her2 monoclonal antibody-producing CHO-K1 cell line (SH87)^13^ was cultivated in PFCDM supplemented with 6 g/l D-(+)-Glucose (Sigma-Aldrich), 8 mM L-Glutamine (Sigma-Aldrich), 0.1% Pluronic F-68 (Life Technologies), and 600 µg/ml G418 disulfate salt (Sigma-Aldrich). Passaging of CHO-K1 and SH87 cells were performed every 3 to 4 days as reported previously^11^.

### Batch cultivation of cells in shake flasks

To determine specific maltose consumption rates in shake flask cultures of non-adapted SH87 and the evaluation of maltose utilization using high initial glucose or maltose concentrations, cells were seeded in PFCDM containing 4 g/l glucose supplemented with 0.5, 1, 2, 3, 10 or 20 g/l maltose, or PFCDM containing 14 or 24 g/l glucose. To compare the growth of CHO-K1 cells with increasing concentrations of maltose, cells adapted to PFCDM maltose medium were seeded into PFCDM maltose medium with 3.6, 10, 20, 30 or 40 g/L maltose at 0.3 × 10^6^ cells/ml in duplicates in single-use Erlenmeyer flasks (Corning). Daily samples were analyzed till culture viabilities were less than 50%.

### Analysis of cell culture samples

Vi-Cell XR Cell Viability Analyzer (Beckman Coulter, Brea, CA) was used to determine viable cell density (VCD) and culture viability according to manufacturer’s instructions. For other parameters, clarified culture supernatant was obtained by centrifugation at 8000 g for 10 minutes prior to analysis. BioProfile 100 Plus (Nova Biomedical, Waltham, MA) was used to analyze ammonium, glutamine, glucose and lactate; Vapor pressure osmometer (Vapro 5520, Wescor, Logan, UT) was used to determine osmolality; Maltose Colorimetric/Fluorometric Assay Kit (Biovision, Milpitas, CA) was used to determine maltose concentration; IMMAGE 800 (Beckman Coulter) was used to quantify monoclonal IgG antibody titer.

### Fed-batch cultivation of cells in bioreactors

For each culture condition to be tested, SH87 was scaled up in PFCDM and inoculated into duplicate 2 liter glass bioreactors (Sartorius, Germany) at a VCD of 3 × 10^5^ cells/ml. Culture temperature, pH, dissolved oxygen and stir rate were maintained at 37 °C, 7.1, 50% and 120 rpm respectively. The culture set points for glucose and glutamine were 0.5 g/l and 0.5 mM respectively, and predictive feeding was used to maintain these concentration levels by the addition of a concentrated DMEM-based protein-free feed and a 150 g/l glucose solution. Daily samples were analyzed till culture viabilities were less than 50%.

### Antibody glycosylation analysis

Antibody glycosylation analysis was performed according to previously published protocol with modifications^14^. Briefly, Protein-A-purified IgG samples were first desalted using a PD 10 column (GE Healthcare, Pittsburgh, PA) following manufacturer’s protocol. Then, glycans were released and labeled with RapiFluor MS (RFMS)^15^ according to manufacturer’s protocol (Waters Corporation, Milford, MA). After labeling, excess RFMS was removed by passing the labeling mixture through a MiniTrap G-10 desalting column (GE Healthcare) and the purified RFMS-labeled glycans were then dried under vacuum. The samples were reconstituted in 200 μl reconstitution buffer containing 42.8 μl of water, 50 μl of dimethylformamide and 107.2 μl of acetonitrile, and analyzed by the UNIFI Biopharmaceutical platform (Waters Corporation, Milford, MA). Raw retention time of each chromatographic peak obtained was converted to a glucose unit (GU) by fitting into a calibration curve established by a RFMS-labeled dextran ladder (Waters Corporation). The observed GU value and the associated mass of each chromatographic peak were then used to search against an experimental database for *N*-glycans embedded in the UNIFI Biopharmaceutical platform, which contains information on expected GU values and masses of more than 300 *N*-glycan species. A structure is then assigned to each chromatographic peak based on two orthogonal criteria: (1) the observed GU value matches the expected GU value within 0.2 GU deviation, and (2) the observed mass matches the expected mass of the glycan within 5 ppm mass error. Additionally, knowledge of CHO glycosylation features was applied as biological filter to remove irrelevant candidate structures, such as glycans with bisecting GlcNAc and α2,6-linked sialic acid.

### Calculations

Specific growth rate (µ) was determined by plotting ln(VCD) vs t (Equation 1). t is culture time, VCD is viable cell density, and VCD_0_ is initial viable cell density.

1 $$\begin{array}{c}{\rm{VCD}}={{\rm{VCD}}}_{0}{{\rm{e}}}^{\mu {\rm{t}}}\\ \mathrm{ln}({\rm{VCD}}/{{\rm{VCD}}}_{0})=\mu {\rm{t}}\end{array}$$

Trapezium rule was used to determine the cumulative integrated viable cell density (IVCD) (Equation 2).

2 $${{\rm{IVCD}}}_{{\rm{t}}}={{\rm{IVCD}}}_{{\rm{t}}-1}+0.5\times {(\text{VCD}}_{{\rm{t}}}+{{\rm{VCD}}}_{{\rm{t}}-1})\times {\rm{\Delta }}{\rm{t}}$$

Specific IgG productivity (q_p_) between culture times t_1_ and t_2_ was determined by plotting IgG titer (P) vs IVCD according to Equation 3.

3 $$\begin{array}{c}{{\rm{q}}}_{{\rm{p}}}=({{\rm{P}}}_{{\rm{t2}}}-{{\rm{P}}}_{{\rm{t1}}})/({{\rm{IVCD}}}_{{\rm{t2}}}\,-\,{{\rm{IVCD}}}_{{\rm{t1}}})\\ {{\rm{P}}}_{{\rm{t2}}}{={\rm{q}}}_{{\rm{p}}}\times ({{\rm{IVCD}}}_{{\rm{t2}}}\,-\,{{\rm{IVCD}}}_{{\rm{t1}}})+{{\rm{P}}}_{{\rm{t1}}}\end{array}$$

Specific substrate consumption rate (q_s_) between culture times t_1_ and t_2_was determined by plotting substrate concentration (S) vs IVCD according to Equation 4.

4 $$\begin{array}{c}-{{\rm{q}}}_{{\rm{s}}}=({{\rm{S}}}_{{\rm{t2}}}-{{\rm{S}}}_{{\rm{t1}}})/({{\rm{IVCD}}}_{{\rm{t2}}}\,-\,{{\rm{IVCD}}}_{{\rm{t1}}})\\ {{\rm{St}}}_{2}=-{{\rm{q}}}_{{\rm{s}}}\times ({{\rm{IVCD}}}_{{\rm{t2}}}\,-\,{{\rm{IVCD}}}_{{\rm{t1}}})+{{\rm{S}}}_{{\rm{t1}}}\end{array}$$

Specific substrate consumption rate (q_s_) was fitted into a Monod model according to Equation 5, where q_s_max_ is the maximum specific substrate consumption rate, S is the substrate concentration, and K_s_ is the affinity constant.

5 $${{\rm{q}}}_{{\rm{s}}}={{\rm{q}}}_{s\_\max }{\rm{S}}/{({\rm{K}}}_{{\rm{s}}}+{\rm{S}})$$

### Data Availability

No datasets were generated or analyzed during the current study.

## Electronic supplementary material

Supplementary Figures
