# Supplementary material for: Application of maltose as energy source in protein-free CHO-K1 culture to improve the production of recombinant monoclonal antibody
Source: Sci Rep. 2018 Mar 6;8:4037. doi: 10.1038/s41598-018-22490-8 (PMC5840386; doi:10.1038/s41598-018-22490-8)
Supplement: Supplementary file 1 — Supplementary Figures [file 41598_2018_22490_MOESM1_ESM.pdf]

Application of maltose as energy source in protein-free CHO-K1 culture to improve the production of recombinant monoclonal antibody

Dawn Sow Zong Leong [+], Brian Kah Hui Teo [+], Janice Gek Ling Tan, Hayati Kamari, Yuan Sheng Yang, Peiqing Zhang, Say Kong Ng *

Bioprocessing Technology Institute, Agency for Science, Technology and Research (A*STAR), Singapore.

[+] These authors contributed equally to the work.

* Corresponding author: Bioprocessing Technology Institute, Agency for Science, Technology and Research (A*STAR), 20 Biopolis Way, #06-01, Centros, Singapore 138668, Singapore. Telephone: 65-64070919, Fax: 65-64789561, Email: ng_say_kong@bti.a-star.edu.sg

Supplementary Figures

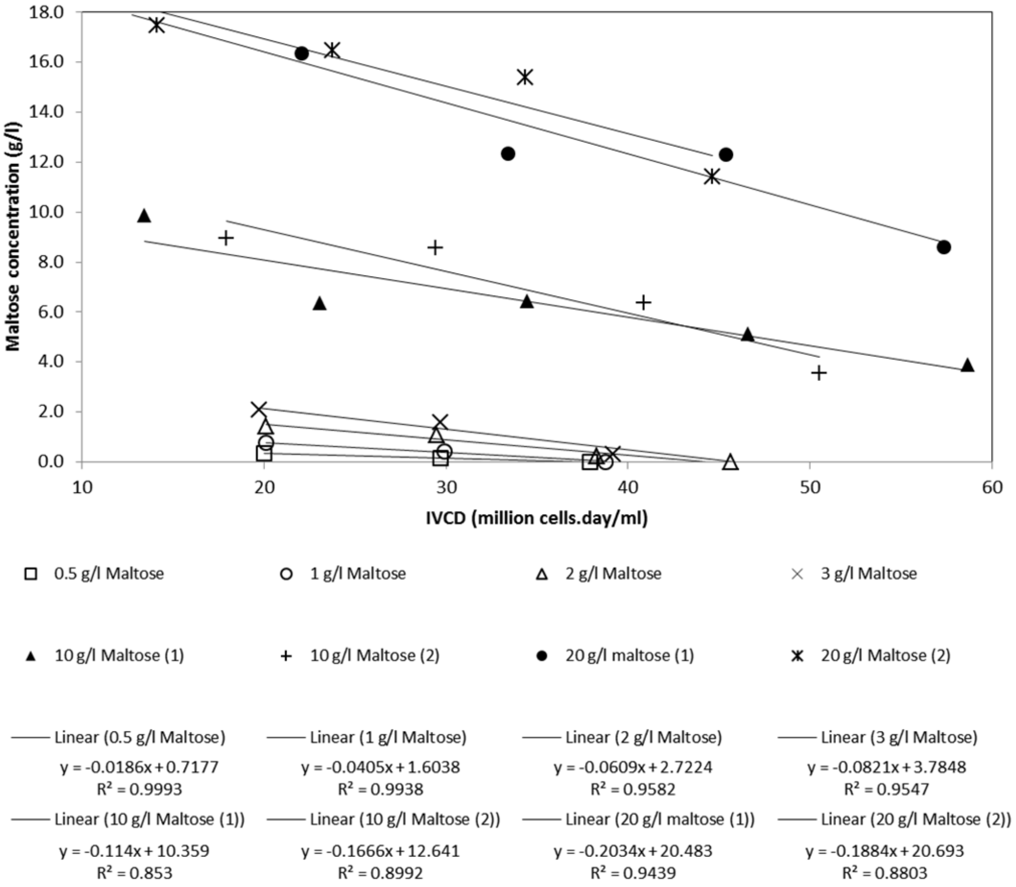

Supplementary Fig. 1. Specific maltose consumption rates of SH87 cultivated in protein-free chemically defined medium (PFCDM). SH87 cell routinely maintained in glucose-only PFCDM was sub-cultivated into PFCDM containing 4 g/l glucose and supplemented with 0.5, 1, 2, 3, 10 and 20 g/l maltose. Maltose concentrations were plotted against IVCD, according to Equation 4. The slope of the graphs gave the specific maltose consumption rates of the cultures over the time periods.

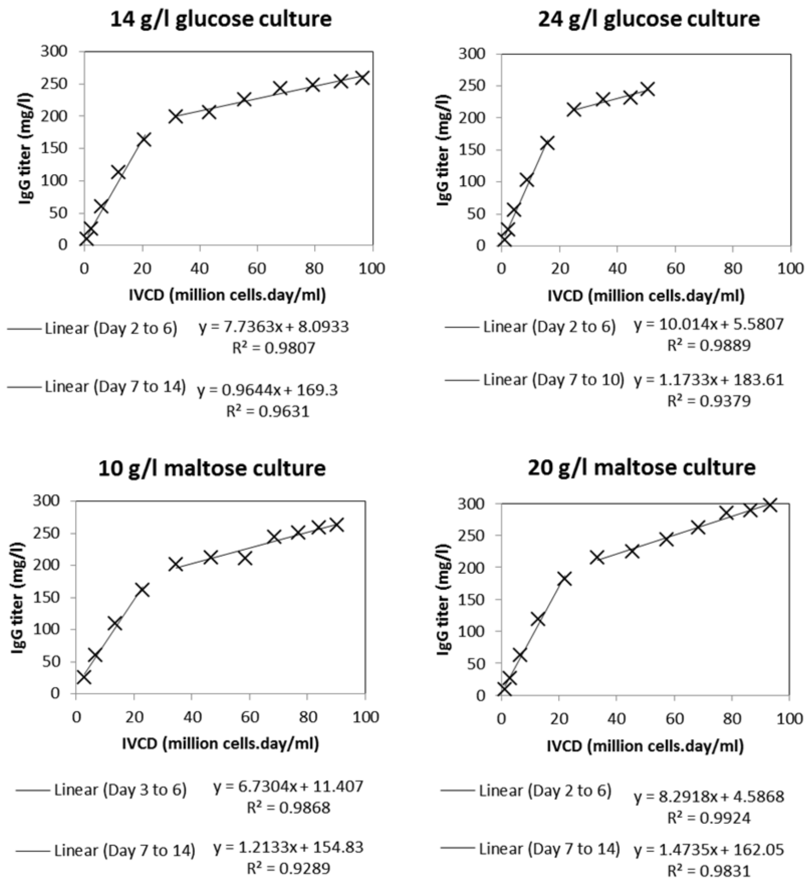

Supplementary Fig. 2. Specific IgG productivities of SH87 cultivated in protein-free chemically defined medium (PFCDM) with high concentrations of glucose and maltose. SH87 cell routinely maintained in glucose-only PFCDM was sub-cultivated into PFCDM with 14 g/l glucose, 24 g/l glucose, 4 g/l glucose + 10 g/l maltose, or 4 g/l glucose + 20 g/l maltose. IgG titers were plotted against IVCD, according to Equation 3. The slope of the graphs gave the specific IgG productivities of the cultures over the time periods.

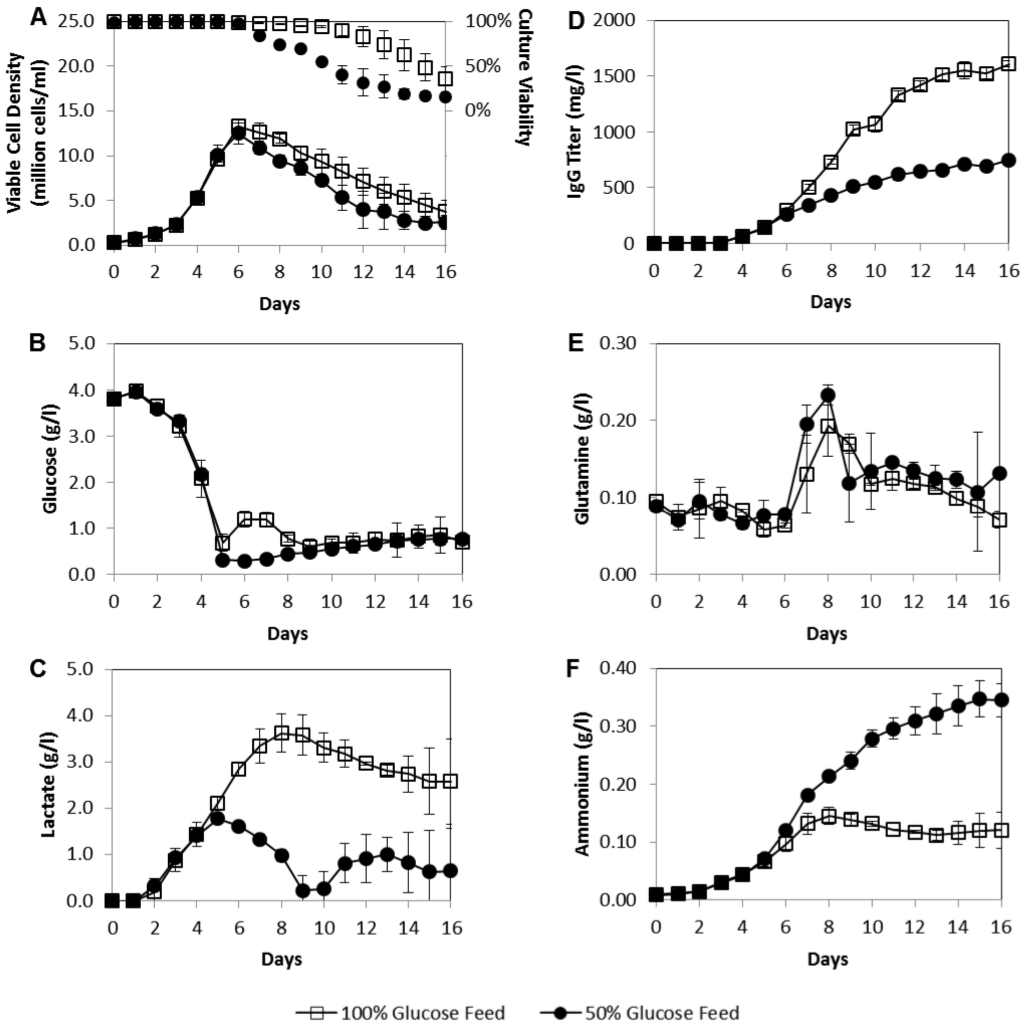

Supplementary Fig. 3. Growth and biochemical profiles of SH87 fed-batch bioreactor cultures in glucose only protein-free chemically defined medium (PFCDM) base medium with glucose fed at 100% or 50% of calculated glucose requirements. SH87 cell routinely maintained in glucose-only PFCDM was sub-cultivated into PFCDM with 4 g/l glucose in 2 liter stirred tank bioreactors. The cultures were fed daily with either 100% of its calculated glucose or with 50% of its calculated glucose requirement. Other nutrients were fed similarly in a separate feed using glutamine as reference nutrient. The cultures were monitored till culture viabilities were lower than 50%, to obtain their (A) Viable cell densities (lined marker) and culture viabilities (marker only), and (B) Glucose, (C) Lactate, (D) IgG titer, (E) Glutamine, and (F) Ammonium profiles. Duplicate bioreactor cultures were performed for each fed-batch condition, and the averages and standard deviations from the replicate bioreactor cultures were plotted. Osmolality maintained below 381 mOsm/kg (data not shown).
